# International Collaboration and Spatial Dynamics of US Patenting in Central and Eastern Europe 1981-2010

**DOI:** 10.1371/journal.pone.0166034

**Published:** 2016-11-15

**Authors:** Balázs Lengyel, Mariann Leskó

**Affiliations:** 1 Department of Civil and Environmental Engineering, Massachusetts Institute of Technology, 77 Massachusetts Avenue, Cambridge 02139, United States of America; 2 International Business School Budapest, Záhony u. 3, 1031 Budapest, Hungary; 3 Centre for Economic and Regional Studies, Hungarian Academy of Sciences; Budaörsi út 45, 1112 Budapest, Hungary; 4 MSc student in Economics, University of Amsterdam, Roetersstraat 11, 1018 WB, Amsterdam, The Netherlands; Charles University, CZECH REPUBLIC

## Abstract

How did post-socialist transition and a parallel shift in international labor division restructure regional innovation systems in Central and Eastern Europe? This question is increasingly important, because current EU innovation policy is combined with regional development in Smart Specialization Strategies; however, spatial trends of innovation in Central and Eastern Europe are not fully understood which might lead to less than perfectly efficient policy. In this paper we describe the spatial dynamics of inventor activity in the Czech Republic, Hungary, Poland and Slovakia between 1981 and 2010 –a period that covers both the late socialist era and the post-socialist transition. Cleaning and analyzing the publicly available data from the United States Patent and Trademark Office we illustrate that Central and Eastern European patents made in international co-operations with partners outside the region receive more citations than those Central and Eastern European patents that lack international co-operation. Furthermore, the technological portfolio of the former patents has become increasingly independent from the technological portfolio of the latter class. A town-level analysis of the applicant-inventor ties reveals that inventors have started to work for foreign assignees in those towns where no innovation activity had been recorded before. However, the positive effect does not last long and patenting seems to be only periodic in the majority of these towns. Therefore, innovation policy in Central and Eastern European countries, as well as in other less developed regions, shall foster synergies between international and domestic collaborations in order to decrease regional disparities in patenting.

## 1. Introduction

The growing scale of international collaboration in knowledge production has been a frequently reported phenomenon since globalization in science and patenting sped up [1, 2, 3]. Scholars also warn us that cross-country co-operation is still weak in areas like the European Union where research integration is an explicit aim [4, 5] and thus suggest policy focusing on international labor division in science and innovation. International collaboration is important in innovation because a greater variety of knowledge can be combined in the invention process when involved parties are from different locations and institutional settings [6, 7, 8, 9]. For example, the number of technological claims and thus the cover of legal protection and the value of the patents are larger in cases of international co-operation compared to domestically-owned patents [10]. Furthermore, international knowledge flows can bring dynamics to domestic innovation and regional development when the knowledge of internationally active agents spills over to co-located firms and inventors [11, 12, 13, 14]. This latter aspect is especially important for less developed countries that can benefit from international collaborations in their knowledge production [15, 16, 17, 18, 19, 20, 21]. Although the territorial dynamics of patenting are often analyzed in developed and also in developing countries [22, 23], very little is known about the effect of international collaborations on the spatial dynamics of knowledge production (e.g. the start and survival of innovation activities in towns).

In this paper we look at the spatial dynamics of patenting at the United States Patent and Trademark Office (USPTO) of four Central and Eastern European (CEE) countries–the Czech Republic, Hungary, Poland, and Slovakia–in the 1981–2010 period on town level. These countries are often referred to as the Visegrad countries and were part of the Eastern Bloc and COMECON before 1991 (the Czech Republic and Slovakia constituted Czechoslovakia at that time). They have gone through a major economic transition from planned economy to market economy in the 1990s and joined the European Union in 2004. The four selected countries have always been lagging behind the average EU15 level in terms of innovation performance; for example, only the best performing CEE country (Czech Republic) could exceed the worst performing EU15 country (Portugal) in 2016 [24]. However, the selected countries produced 3 times more USPTO patents altogether over the investigated period than the rest of CEE transition economies (based on information described in Section 2.1).

Our historical case is particularly interesting, because the radical political and economic turn was followed by a sharp fall in innovation activities in the early 1990s mainly because R&D-intensive state-owned companies were either closed down or got privatized. The latter process resulted in a thorough portfolio-cleaning [25, 26, 27]. Globalization gathered speed simultaneously, opening up new possibilities of international collaborations for CEE researchers but foreign control has increasingly dominated patenting, posing a riddle for national and regional policies [3]. The question how foreign-controlled innovation should be handled in CEE is still not clear. On the one hand, international R&D collaborations embodied in foreign-owned patents can be very important sources of new knowledge that can spill over to domestic firms [15, 16]. On the other hand, foreign firms can crowd out domestic firms by taking over too much of the innovation capacities [28, 29]. Although large efforts have been devoted to strengthen regional and national innovation systems in CEE after the countries joined the EU [30, 31], there is a common agreement that innovation policy could not cope with its duties due to weak local institutions and poorly developed innovation links between local actors [32, 33, 34, 35, 36, 37]. More recently, the efforts of EU innovation policy and cohesion policy are combined in the Smart Specialization Strategies, which is mostly based on best practices of EU15 regions [38, 39, 40]. However, the lack of deep understanding of CEE trends could lead to less efficient policy and therefore, further research is needed.

To contribute to the policy-related discussion, we outline three major trends of collaboration of CEE inventors with non-CEE and CEE firms in patenting. The paper has a descriptive nature; we demonstrate various associations in the data but do not aim to explore the causal relationship between international R&D collaborations and domestic innovation. We collect information about those USPTO patents that contained at least one CEE inventor over the investigated period and test three hypotheses formulated on the basis of the above literature.

Hypothesis 1 (*H1*): *The USPTO patents assigned to non-CEE firms and invented or co-invented by at least one CEE inventor receive more citations than USPTO patents assigned to CEE firms and invented or co-invented by at least one CEE inventor*. The rationale behind *H1* is the positive association between international collaboration and other patent quality indicators [10, 12]. Although criticized in the literature [10] the number of citations has been frequently used to predict patent quality [41, 42, 43]. Another reason to choose this indicator is that it is easier to access than other types of measurement. The verification of *H1* would imply that policy should foster international collaborations in patenting because participating inventors can learn from these projects.

However, the question whether the knowledge of these inventors can spill over to other co-located inventors is less clear [11, 15, 16] because brain-drain from domestic to foreign firms can reduce the absorptive capacity of domestic R&D [28]. Furthermore, the technological distribution of foreign- and domestically-controlled innovation can be very different, which can also hinder the effect of knowledge spillovers because CEE inventors active in international projects might gain experience in very different fields than domestic CEE inventors work in [27]. Therefore, we have to better understand if foreign-controlled patents have restructured CEE innovation over the post-socialist transition similarly as it was shown by using other type of R&D data [29]. Hypothesis 2 (*H2*): *There is a significant difference between the technological distributions of the group of patents invented or co-invented by at least one CEE inventor and assigned to non-CEE firms and the group of patents invented or co-invented by at least one CEE inventor and assigned to CEE firms*.

Finally, we test the effect of international collaboration on the spatial dynamics of CEE patenting, which might be important because regions might benefit from the access to external R&D funds and thus produce more innovation [6, 7, 8]. On the contrary, inventors might also take advantage of geographical proximity and shared institutional background when collaborating with domestic firms [7, 11]. In order to gain a better understanding, we look at the start and survival rate of invention activities in CEE towns depending on the two types of collaborations. Hypothesis 3 (*H3*): *The collaboration of CEE inventors with non-CEE assignees increases the likelihood that patenting appears and survives in towns*, *as opposed to the collaboration with CEE assignees*.

## 2. Materials and Methods

### 2.1 Data

Using techniques for USPTO data collection and organization developed recently by [44, 45, 46], we have downloaded the full set of patents, in which at least one inventor participated from the Czech Republic, Poland, Slovakia, and Hungary between 1981 and 2010 on August 5, 2013. USPTO data was used instead of European Patent Office (EPO) data because the accession of CEE countries into the common EU market may have affected the number of EPO patent applications for reasons other than inventions [47]. Also, USPTO patents can be expected to capture globally competitive innovation output better than EPO data [48, 49].

The download retrieved 5,777 patents. The data includes the name and address of inventors and assignees and the number of citations the patent received until the date of download. The dataset also contains the full codes for technological fields according to Cooperative Patent Classification (CPC) that is the harmonized classification system based on the existing former classifications of ECLA (European Classification) and USPS (United States Patent Classification). One can find detailed description of the classification system at http://www.cooperativepatentclassification.org.

This was followed by a thorough cleaning process concerning the technological field of patents, the name of assignees and CEE inventors and the name of the town of assignee locations and CEE inventor home addresses. We had to exclude those patents that could not be cleaned. As a result, the data contains 5,078 patents from 1,570 assignees located in 47 countries and 11,405 inventors located in 57 countries. In the next step, we identified the geo-coordinates of assignees and CEE inventors based on the cleaned names of towns. In the last step, we matched NUTS3 region codes and population sizes to every CEE town in our data from a publicly available EUROSTAT database that one can assess at http://ec.europa.eu/eurostat/web/nuts/correspondence-tables/postcodes-and-nuts.

We provide further information on data collection, cleaning and patent exclusion criteria in S1 Appendix. The cleaned dataset that contains all necessary information for the analysis can be retrieved from http://datadryad.org/review?doi=doi:10.5061/dryad.5c820.

### 2.2 Methods

In order to test *H1*, we compared the total number of citations of patents assigned to non-CEE firms to patents assigned to CEE firms by using two methods. First, we binned the data into 5-year periods and applied the *U*-test (see also as Wilcoxon-Mann-Whitney test) for each period. This method is a non-parametric analog of the *t*-test but we do not have to assume that the dependent variable is normally distributed, which is very important because citation distributions are typically strongly skewed to the right. If the null hypothesis is verified, the case that a patent assigned to non-CEE firms exceeds a patent assigned to CEE firms in terms of total number of citations has equal probability to the contrary case when the number of citations of patents assigned to CEE firms is higher. A significant test would reject the null hypothesis and the comparison of rank sum values to the expected values can enable one to detect which distribution is greater. Second, we visualized the distribution of citations of patents in both groups and for the full 1981–2010 period on a log-log scale and checked whether one fitted curve could describe both distributions.

For testing *H2*, we compared the technological distribution of patents assigned to non-CEE firms to patents assigned to CEE firms and tested the independence of the categorical variables of technological class versus the type of assignees with Pearson’s chi-squared test and the Fisher’s exact test. The inclusion of the latter test is important if we want to assess the independence of the variables over time because splitting the data leads to cells with low expected values. We performed the tests on the basis of the full 1981–2010 period and on a 5 year and 1 year basis as well in order to understand the dynamics of technological change. A significant result would suggest a dependent relationship between the type of assignees and the technological classification of patents.

Finally, we binned the data into 5-year periods and aggregated the inventor-assignee links to the town level for mapping purposes and illustrated the change in the spatial patterns of domestic and international collaboration in CEE patenting. Then, we constructed a panel of CEE towns where at least one inventor was found over the full 1981–2010 period and ran two types of pooled probit regressions to test *H3*. First, the binary dependent variable is *ENTRY* that is only equal to 1 at period *t* if at least one inventor resides in the town at period *t* but not at *t*-1 and 0 otherwise. Second, the binary dependent variable *EXIT* is only equal to 1 at period *t* if at least one inventor resides in the town at period *t*-1 but not at *t* and 0 otherwise. For example, if inventors reside in the CEE town only in periods 1986–1990, 1991–1995, and 2001–2005; *ENTRY* is equal to 1 in periods 1986–1990 and 2001–2005, while *EXIT* is equal to 1 in periods 1991–1995 and 2001–2005.

We used dummy variables to estimate the effect of international collaborations on the likelihood that patenting starts and survives in CEE towns in comparison to domestic collaboration. The indicator *NONCEE*_*it*_ takes the value of 1 if the inventors in town *i* worked solely for non-CEE assignees at period *t* and 0 otherwise. Similarly, the variable *CEE*_*it*_ takes the value of 1 if the inventors in town *i* worked solely for CEE assignees (be the assignees located in identical or in other CEE towns) at period *t* and 0 otherwise. The baseline category of the regression is the group of those CEE towns where inventors cooperate with both non-CEE and CEE inventors at period *t*, which is mutually exclusive with the above two groups. Significant point estimates would suggest that starting and finishing innovation activities have significantly different probabilities in the above defined groups than in the baseline group. In order to track and compare these probabilities over time, we introduced period fixed effects that are interacted with the above explanatory variables. Significant estimates of the interaction term would suggest significant change of the explanatory variables over time. The formula of the estimation is given by

$$Y_{it}^{*}=\beta_{1}{CEE}_{it}+\beta_{2}{NONCEE}_{it}+\beta_{3}{CEE}_{it}\times T_{t}+\beta_{4}{NONCEE}_{it}\times T_{t}+{\gamma D}_{i}+\delta {POP}_{i}+{\theta T}_{t}+\varepsilon_{it},$$

where

$$Y=\left\{ \begin{array}{l} 1\;if\;Y^{*}>0\\ 0\;otherwise. \end{array}\right.$$

*D*_*i*_ denotes a combination of country and regional dummies. Country dummies are used in order to control for institutional differences and also for deviation in spatial dynamics across CEE countries. Further regional dummies reflecting the NUTS3 regions of European classification are used to control for unobserved regional differences within countries (e.g. R&D infrastructure). *POP*_*i*_ refers to the log-transformed value of population of town *i* in year 2010 that is used to control for the type of towns; and *T*_*t*_ refers to time fixed-effects. The point estimates and standard errors were calculated by the maximum likelihood method.

## 3. Results

The results of the paper are divided into two parts. In the first step, we describe the trend of international collaboration and the share of foreign assignees; illustrate how internationally collaborative patents differ from domestic patents in terms of number of citations and technological profile and test *H1* and *H2*. This is followed by a geographic investigation of assignee-inventor ties on the town level, in which we test *H3*.

### 3.1 International collaboration, impact and technological profile of CEE patenting

**Fig 1:**
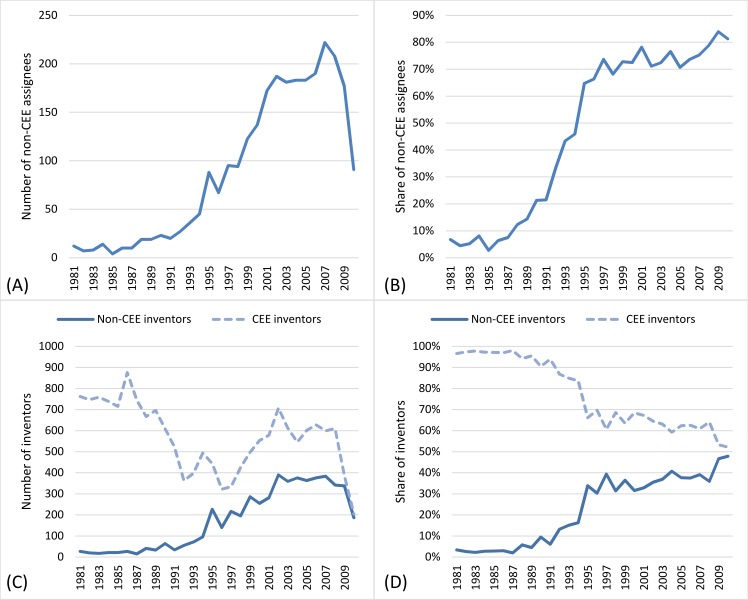
Non-CEE assignees and inventors working with CEE inventors, on yearly basis, 1981–2010. (A) Number of non-CEE assignees weighted by the number of patents filed by them. The result is identical when using the non-weighted raw number of individual assignees. The sharp decline in 2009 is not the result of data cleaning and might be due to the temporal drop related to the post-2007 financial crisis as it was demonstrated in other cases [50, 51]. (B) Share of non-CEE assignees. The ratio of non-CEE assignees are only slightly more than 5 percent in 1981 and reach more than 80 percent at the end of the period. (C) The number of CEE- and non-CEE inventors authoring CEE patents weighted by the number of authored patents. (D) Share of non-CEE and CEE inventors authoring CEE patents weighted by the number of authored patents.

Fig 1A and Fig 1B illustrate a significant acceleration of international co-operations between CEE inventors and non-CEE assignees over the 1990s. This may be associated with the regime change in the post socialist countries, when markets became more open and thus, working with assignees from other countries became accomplishable. The high share of non-CEE assignees found here supports the idea [28] that international collaboration dominates innovation in CEE countries to a larger extent than in more developed innovation systems. Furthermore, CEE inventors not only worked for a growing number of non-CEE assignees, but collaboration with non-CEE inventors became very important as well. Fig 1C illustrates that the number of CEE inventors fell dramatically from the middle 1980s and only started to rise again in the mid-1990s. Meanwhile, the number of non-CEE co-inventors grew over the 1990s, and the acceleration only slowed down in the 2000s, when the ratio almost reached 50 percent (Fig 1D). These illustrations are based on yearly distributions because the number of observations does not allow for the aggregation for longer periods.

**Fig 2:**
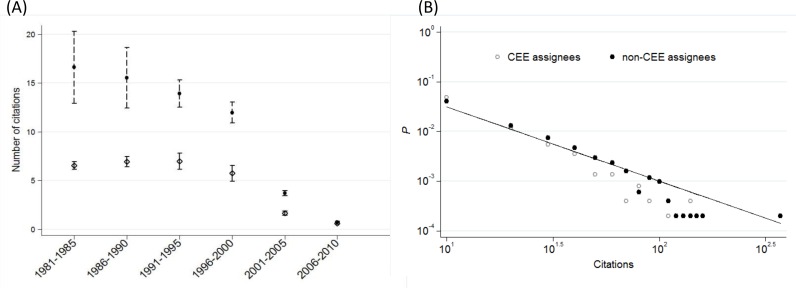
Citation distributions of patents assigned to CEE and non-CEE firms. (A) The mean and standard deviation of citations per patent. Black dots and dashed ranges depict the mean and standard deviation of patents assigned to non-CEE firms. Hollow diamonds and solid ranges depict the mean and standard deviation of patents assigned to CEE firms. (B) Probabilistic distribution of citations on log-log scale, 1981–2010. Citations of CEE and non-CEE patents were binned into 10^1^ intervals for *P* calculation. The slope of the solid line is -1.5.

In order to illustrate the difference in the number of citations between patents assigned to CEE firms and patents assigned to non-CEE firms, we binned the distributions into 5-year intervals to avoid the problem of low numbers; calculated the mean and standard deviation and compared them in Fig 2A. Naturally, the average citation falls near the end of the period, since old patents had more time to be discovered and cited than the young ones. With having this in mind, we observe that the patents of non-CEE assignees are at least two times more cited on average than the patents of CEE assignees.

However, the citations are not normally distributed in either groups and can be better described by a power-law (Fig 2B), which is typical for a variety of empirical data, including patents [52, 53, 54]. One can observe a slightly higher probability of CEE patents at the lowest value interval but the *P* value is higher for the non-CEE group in almost all other intervals. This suggests that the negative exponent is smaller in the case of non-CEE patents. Indeed, the solid line fitted to the medium values of the non-CEE distribution by hand has a slightly higher fit (R^2^ = 0.34) than the one fitted to the CEE patents’ distribution (R^2^ = 0.25).

In a next step, we binned the data into 5-year periods to collect enough observations and applied *U*-test to assess if there was significant difference across the above distributions; results are reported in Table 1. The *P* values are below 0.001 in the majority of periods and it is 0.012 in the 2005–2010 period. Based on recognized standards of statistical significance, we can reject the null hypothesis of identical distributions. The comparison of rank sum and expected values by types of assignee confirms that the citations of patents owned by non-CEE firms are higher in every period than the citations of patents owned by CEE firms. Therefore, *H1* is verified. The result suggests that international co-operation results in a better quality of invention, if one accepts the number of citations as an indicator of quality [41, 42, 43].

**Table 1:** Citations of patents by type of assignee, U-test

	1981–1985		1986–1990		1991–1995		1996–2000		2001–2005		2005–2010	
Type of assignee	Non-CEE	CEE	Non-CEE	CEE	Non-CEE	CEE	Non-CEE	CEE	Non-CEE	CEE	Non-CEE	CEE
Observations	45	762	81	602	216	275	516	210	906	325	888	252
Rank sum	24,700	301,327	34,704	198,882	62,767	58,018	199,201	64,699	599,225	159,070	515,139	135,230
Expected	18,180	307,848	27,702	205,884	53,136	67,650	187,566	76,335	558,096	200,200	506,604	143,766
*z*	4.316		4.222		6.201		4.563		7.792		2.493	
*P* >|*z*|	<<0.001		<<0.001		<<0.001		<<0.001		<<0.001		0.0127	

In order to evaluate whether technological portfolios are different, we compared the distribution of patents across the main categories of Cooperative Patent Classification (CPC) and by assignee type over the full 1981–2010 period in Table 2. Although there can be overlaps at lower levels of CPC aggregation, Pearson’s chi-squared test reveals that the technological distributions of CEE and non-CEE assigned patents are independent from each other. Therefore, *H2* is verified.

**Table 2:** Technological distribution of patents by assignee type.

CPC Technology class	Assignee type		
	Non-CEE	CEE	
A	385	550	
	(488.3)	(446.7)	
B	213	292	
	(263.7)	(241.3)	
C	547	886	
	(748.4)	(684.6)	
D	130	148	
	(145.2)	(132.8)	
E	10	42	
	(27.1)	(24.8)	
F	150	171	
	(167.6)	(153.4)	
G	603	217	
	(428.2)	(391.8)	
H	614	120	
	(383.3)	(350.7)	
Total	2.652	2.426	
Pearson’s chi-squared (7)	649.3081	
*P*	<<0.001	

Note: Expected values under the validity of the null hypothesis in parantheses. The number of non-CEE patents is higher than the expected value in the case of G and H classes.

**Fig 3:**
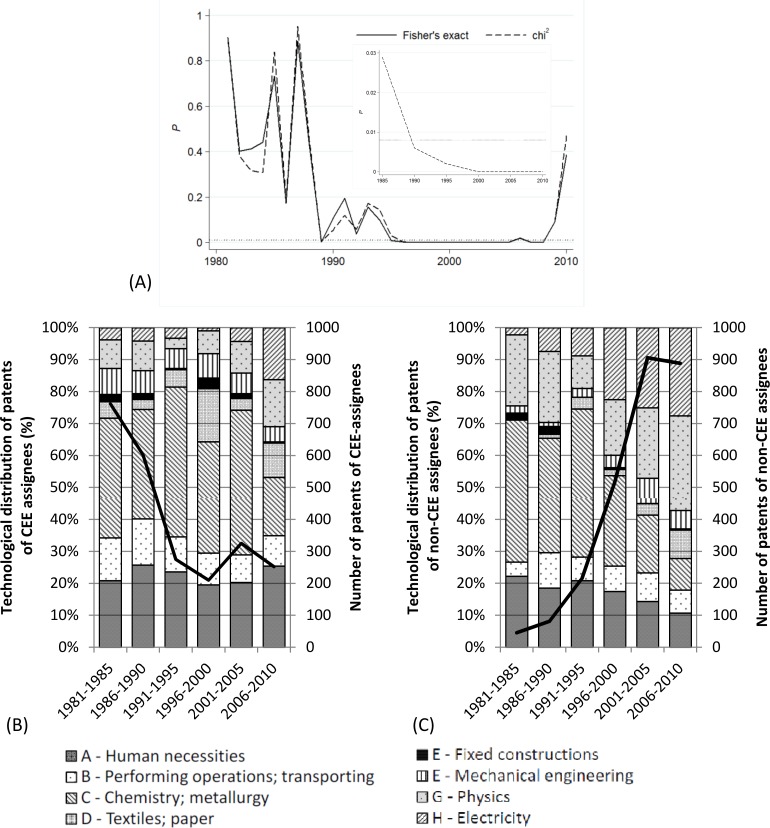
Technological distribution of patents by type of assignee. (A) *P* values of independence tests. The nested figure illustrates the test run on 5-year periods; the framing figure illustrates test results on a yearly basis. The solid line represents Fisher’s exact test; the dashed line denotes Pearson’s chi-squared test and the red dotted line depicts the significance level after Bonferroni corrections. (B) The number and technological distribution of patents owned by CEE assignees. Chemistry and metallurgy and Human necessities dominated patenting of domestic CEE firms in the socialist era and these CPC classes did not lose dominance in the post-socialist transition either. (C) The number and technological distribution of patents owned by non-CEE assignees. Chemistry and metallurgy and Human necessities have been an important field of the widening cooperation with non-CEE assignees. However, most of the patents filed by non-CEE firms starting from the 2000s were classified into Electricity and Physics. These two categories were present in CEE patenting over the entire examined period but their shares stayed quite low throughout.

In a further step, we tested the independence of the above distributions over time. We first binned the data into 5-year periods and calculated chi-squared for every period. Fig 3A demonstrates that *P* values are below 0.008 (the significance level after Bonferroni correction) in all but the first period, which is further evidence of the independence of the distributions. To get an even closer picture, we repeated the exercise on a yearly basis. Besides the chi-squared test, here we applied Fisher’s exact test as well because the yearly samples contain cells with a very low number of observations, which might distort the level of significance in the chi-squared test. Fig 3A illustrates that *P* values of the two methods strongly correlate. Interestingly, one can find no independent technological distributions of Non-CEE and CEE patents in the 1980s because the large *P* values do not allow us to reject the null hypothesis. The higher values of overlap are in line with our expectations because it might have been difficult for CEE inventors to get engaged in international collaboration in the socialist era and therefore these collaborations could be solely based on domestic capacities. However, regardless of data aggregation, the independence of foreign-controlled patenting from domestic CEE patenting from the mid-1990s until the mid-2010s holds.

S2 Appendix contains a table with the exact number of patents by technological classes, 5-year periods, and types of assignees and provides further details regarding the significance of technological change over time.

The findings concerning the post-socialist transition suggest that international collaboration led to a shift in the technological profile of CEE inventors and support the idea that the overlap is small between the innovative capacities controlled by foreign and domestic firms. However, the interesting results regarding the last period need to be addressed by further research because our findings can be attributed to coincidence, an emerging co-evolution of foreign and domestic control, or a mixture of these two.

### 3.2 Inventor-assignee links and spatial dynamics

**Fig 4:**
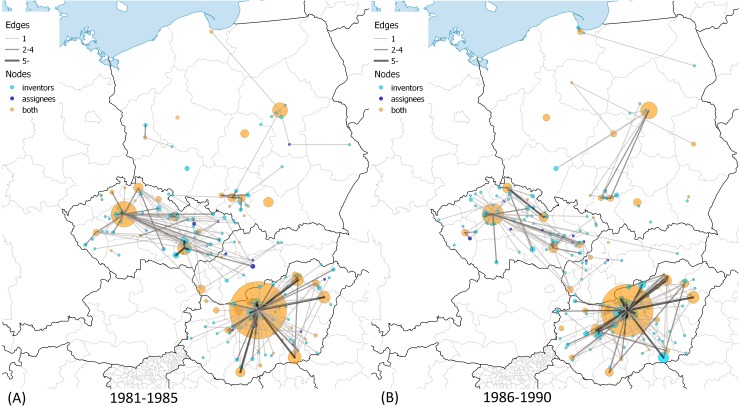
Assignee-inventor links between towns in CEE countries, 1981–1990. (A) 1981–1985. The largest CEE innovation centers are Budapest, HU with 475; Prague, CZ with 100; Warsaw, PL with 33; Brno, CZ with 26; and Szeged, HU with 26 patents. (B) 1986–1990. The largest CEE innovation centers are Budapest, HU with 397; Prague, CZ with 87; Warsaw, PL with 41; Dunakeszi, HU with 22; and Debrecen, HU with 21 patents. Own work with Natural Earth base map (free vector and raster map data). Cartography licensed under CC BY-SA 4.0.

**Fig 5:**
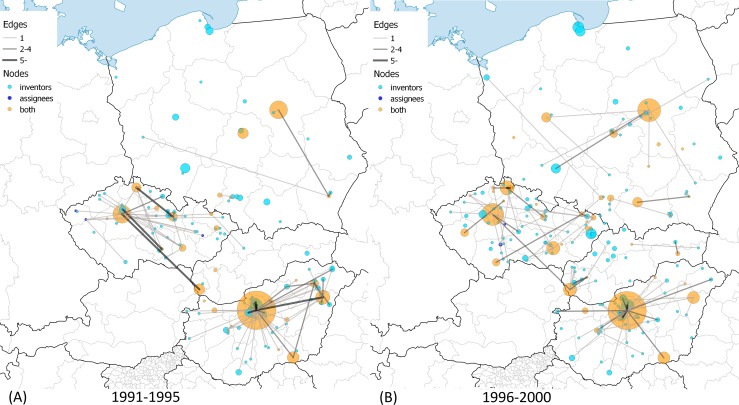
Assignee-inventor links between towns in CEE countries, 1991–2000. (A) 1991–1995. The largest CEE innovation centers are Budapest, HU with 214; Prague, CZ with 59; Warsaw, PL with 46; Debrecen, HU with 29; and Dunakeszi, HU with 20 patents. (B) 1996–2000. The largest CEE innovation centers are Budapest, HU with 210; Prague, CZ with 97; Warsaw, PL with 76; Liberec, CZ with 29; and Bratislava, SK with 25 patents. Own work with Natural Earth base map (free vector and raster map data). Cartography licensed under CC BY-SA 4.0.

**Fig 6:**
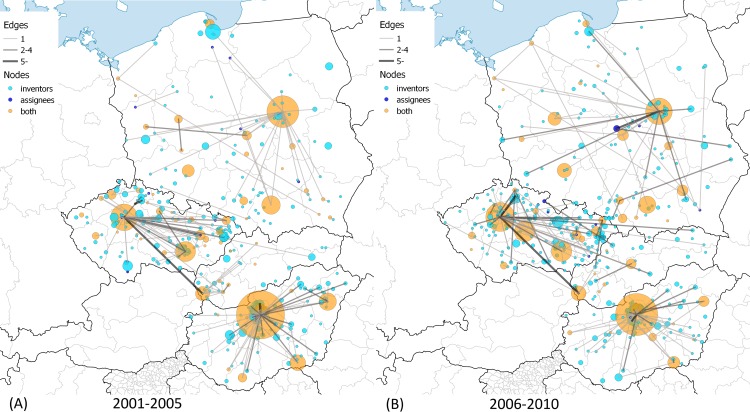
Assignee-inventor links between towns in CEE countries, 2001–2010. (A) 2001–2005. The largest CEE innovation centers are Budapest, HU with 324; Warsaw, PL with 141; Prague, CZ with 127; Brno, CZ with 55; Cracow, PL with 47. (B) 2006–2010. The largest CEE innovation centers are Budapest, HU with 243; Prague, CZ with 141; Warsaw, PL with 96; Hroznetin, CZ with 60; and Brno, CZ with 56 patents. Own work with Natural Earth base map (free vector and raster map data). Cartography licensed under CC BY-SA 4.0.

A set of maps were drawn in order to illustrate the spatial dynamics of CEE patenting binned into six 5-year periods in Fig 4, Fig 5 and Fig 6. In order to show the dynamics of assignee-inventor collaboration of CEE towns in space, we categorized the towns into three classes. Nodes depict those towns where (1) only inventors (light-blue), (2) only assignees (dark-blue), and (3) both inventors and assignees were located (orange) in the given period. The size of the nodes indicates the number of patents filed by inventors living in the given town in the case of light-blue and orange nodes. It is important to compare these two types of towns with the dark-blue nodes, the sizes of which are determined by the number of patents filed by assignees. If at least one patent was filed in collaboration between an inventor in town A and an assignee in town B, then there is a link between towns A and B. The thickness of the edges depicts the number of patents filed as it is indicated in the legend of the maps.

One can make a few important observations when examining the maps. Not only the spatial distribution and dynamics of inventors and assignees in CEE countries but also the spatial dynamics of their collaboration can be described.

The distribution of orange nodes suggests that patenting is concentrated in agglomerations of capital cities and regional centers like university towns. However, there is a considerable difference regarding the above statement across CEE countries, which is especially true after 2001. Hungarian major university towns could not increase the volume of patenting and catch up to the Budapest agglomeration; meanwhile one can observe that regional centers emerged in the Czech Republic and Poland. Cross-country differences prevail in terms of the light-blue nodes as well. Hungarian inventors are concentrated with a growing intensity in the Budapest agglomeration, while the spatial distribution of inventors in the Czech Republic and Poland became more equal over time. Slovakia had very few towns that were active in US patenting over the period, but a small agglomeration around Bratislava emerged in the late 1990s.

Collaboration with partners from other CEE countries was rare. The only exceptions were the co-operation links between Slovakian inventors and Czech assignees before 1990. The majority of these collaborations disappeared after the cessation of Czechoslovakia despite the strong link between Prague and Bratislava. However, the maps contain many small inventor towns and a few regional centers as well–like the Gdansk area in 1991–1995 and 1996–2000 –that have no connections in the map. The inventors in these towns co-operated with assignees located in foreign countries and not in CEE. The amount of these towns grew continuously over the full period. As we illustrated above, international collaboration intensified, due in large part to the strengthened collaboration with assignees in US cities. S1 Fig and S2 Fig visualize the global map of town-level collaboration in CEE patenting.

Table 3 provides additional descriptive information of the assignee-inventor town-level networks. The number of edges grew over the period, which is not true for edges across CEE towns. The number of the towns where assignees and inventors are found as well fell in the early 1990s and then rose back to the level of the 1980s only after 2000. In contrast, the number of the towns with inventors more than doubled after 1995. The growth is true for non-CEE assignee cities as well, which evidently accords with the emergence of non-CEE edges.

**Table 3:** The global network of CEE patenting.

Period	1981–1985	1986–1990	1991–1995	1996–2000	2001–2005	2006–2010
Edges	277 (332)	315 (360)	279 (308)	442 (482)	809 (860)	770 (808)
Edges in CEE	242 (297)	249 (294)	111 (140)	121 (161)	195 (246)	172 (210)
Inventor towns in CEE	154	165	112	170	338	367
Assignee towns in CEE	12	11	4	3	11	11
Towns with inventors and assignees in CEE	60	50	29	44	64	64
Non-CEE assignee towns	25	52	98	178	237	199

Note: Town-level self-loops, when the inventor and assignee of the patent are located in the same town, are in parenthesis.

International collaboration might be an important source of spatial dynamics. To provide a descriptive illustration about the entries in Table 4, we define a town ENTRY at period *t* if at least one inventor resides in the town at period *t* but not at *t*-1. A majority of the towns that started patenting in a given 5-year period were only linked to other CEE towns in the 1980s and the 1990s as well. However, inventors in most of the entering towns worked only for non-CEE assignees in the 2000s.

**Table 4:** The probability of town entry by the type of international collaboration.

Period	1986–1990	1991–1995	1996–2000	2001–2005	2006–2010
Number of ENTRY	116	67	145	279	236
CEE	95%	61%	53%	36%	37%
NONCEE	3%	37%	42%	54%	60%
CEE and NONCEE	2%	1%	5%	10%	3%

In Table 5, a town is defined EXIT at period *t* if at least one inventor resides in the town at period *t*-1 but not at *t*. The town is INCUMBENT at period *t* if at least one inventor resides in the town at period *t*-1 and then at *t* as well. One can observe that CEE and non-CEE towns have almost equal EXIT rates in 2001–2005. However, the vast majority of the towns where inventors worked for both CEE and non-CEE firms continue patenting.

**Table 5:** The probability of town exit by the location of assignees.

Period	1985–1990	1991–1995	1996–2000	2001–2005	2006–2010
Number of CEE	199	191	80	101	129
INCUMBENT	43.2%	30.4%	37.5%	47.5%	37.2%
EXIT	56.8%	69.6%	62.5%	52.5%	62.8%
Number of NONCEE	5	6	37	74	202
INCUMBENT	60%	16.7%	48.6%	54.1%	36.6%
EXIT	40%	83.3%	51.4%	45.9%	63.4%
Number of CEE and NONCEE	10	18	24	39	71
INCUMBENT	100%	83.3%	87.5%	89.7%	80.3%
EXIT	0%	16.7%	12.5%	10.3%	19.7%

In order to test *H3*, we ran the probit regression specified in section 2.2. We ran the regression separately on a balanced panel of inventing towns for dependent variables *ENTRY* (Models 1–3) and *EXIT* (Models 4–6) and for three time periods. Results are summarized in Table 6.

**Table 6:** Spatial dynamics of patenting, cross-sectional probit regression.

	*ENTRY*	*EXIT*
*CEE*	3.070***	2.604***
	(0.208)	(0.184)
*NONCEE*	2.750***	2.523***
	(0.188)	(0.166)
*CEE* × period 1981–1985		4.903***
		(0.275)
*CEE* × period 1986–1990	0.192	1.140***
	(0.307)	(0.287)
*CEE* × period 1991–1995	0.362	0.921***
	(0.418)	(0.303)
*CEE* × period 1996–2000	0.420	0.470
	(0.292)	(0.305)
*CEE* × period 2001–2005	-0.422	
	(0.260)	
*NONCEE* × period 1981–1985		4.847***
		(0.699)
*NONCEE* × period 1986–1990	0.294	1.085*
	(0.623)	(0.635)
*NONCEE* × period 1991–1995	1.114**	0.766**
	(0.446)	(0.353)
*NONCEE* × period 1996–2000	1.122***	0.415
	(0.301)	(0.304)
*NONCEE* × period 2001–2005	-0.243	
	(0.231)	
pseudo R-sq	0.615	0.613
N	5,136	4,340

Note: Additional control variables are town population, region dummy, country dummy and period dummy. The reference category in the period fixed effects is the 2006–2010 interval in the *ENRTY* model and the 2001–2005 interval in the *EXIT* model; the use of other intervals provide similar results. Missing coefficients are due to collinearity and omitted variables. Standard errors in parentheses.

* p<0.10

** p <0.05

*** p<0.01. Wald test suggests that all coefficients are different from zero.

The estimates of *CEE* and *NONCEE* variables can be interpreted as the effect of the independent variables on the probability of *ENTRY* and *EXIT* in a comparison to the baseline category. This latter baseline category is mutually exclusive with the explanatory categorical variables and takes the value of 1 if the inventors in the town work for both CEE and non-CEE assignees as well in time *t* and zero otherwise. The application of such a baseline category is reasonable because those large towns where inventors work for both domestic and foreign firms are constantly patenting and do not enter or exit the data.

The *ENTRY* model implies that local CEE collaboration induced the probability that a new town begins patenting more than international collaboration did over the full 1981–2010 period. However, the difference between the two main effects is not significant and one can only observe divergence in the interaction terms. We find that none of the *CEE*-period interactions are significant, and thus we find no significant changes in the effect of domestic collaboration over time. The significant coefficients of *NONCEE*-period interactions mean that the effect of international collaboration is significantly stronger in the 1991–1995 and 1996–2000 periods than in the baseline period, which suggests that international collaboration gained importance in the 1990s. The *EXIT* model reveals an even more crucial finding: international collaboration has no long-lasting footprint on regional patenting. The positive and significant coefficients of the main effects mean that both town categories are more likely to exit than the baseline category. Moreover, the coefficients do not differ significantly from each other suggesting that the towns where the inventors worked for non-CEE assignees only, are equally likely to stop patenting as the ones where inventors worked solely for CEE assignees. Further, one can observe a very similar pattern in the interaction terms as well, which implies that international collaboration does not support the survival of patenting in CEE towns. Therefore, we have to reject *H3*. Logit and ordinary least square regression models with identical variables have been run to check the robustness of the findings, which did not change the interpretations of the results.

In summation, we find that international collaboration inevitably became a major engine for spatial dynamics of US patenting in CEE, but its effects are not long-lasting, which calls for policy intervention.

## 4. Discussion

In this paper, we carefully cleaned and analyzed the publicly available USPTO data for the Czech Republic, Hungary, Poland, and Slovakia over the 1981–2010 period and focused on international patent collaborations in order to draw consequences regarding regional development and innovation policy. Our case is interesting because urban scaling was found to be more intensive in CEE than in EU15 countries [55] suggesting that big cities converge quickly to the European trend but peripheral locations in these countries do not. Although it is questionable how innovation plays a role in the above process, the case of Portugal shows us that the lack of innovation hinders the chance for long run convergence [56]. Other examples from peripheral areas including New Zealand [15], Norway [20] and Sweden [19] highlight the importance of interregional and international collaboration. Because the innovation infrastructure is poorly developed in CEE locations, innovative firms build extensively on sources located elsewhere and thus, are more active in interregional and international collaboration. One might also argue that international collaboration is an important source of knowledge spillovers in less developed innovation systems [17], such as the ones in CEE, because inventors can learn from their foreign partners–especially when they participate in high impact innovation–and might transfer new knowledge to their domestic peers.

However, our results imply that there is a very low chance of local knowledge diffusion derived from international patent collaborations. In line with previous literature, we found that international collaborations produced better patents in terms of received citations [10] but also illustrated that the growing scale of international collaborations were associated with a shift in the technological portfolio of CEE innovation [27]. The shrinking overlap between international and domestic innovation is shocking because it is hard to imagine the knowledge transfer between very different technological fields. A well established argument in the literature claims that shared technological background is necessary for learning [7] and thus diverging technological portfolios of domestic and international collaboration decrease the probability of knowledge spillovers in CEE.

Another important observation of our study is that due to international collaborations CEE inventors started to patent in towns where no patenting activity has been documented before as well and this effect has been increasing over time. This is an important trend, because patenting activity fell sharply in CEE over the post-socialist transition [25, 26], from which regions with an inflexible industrial structure suffered the most [28]. International collaboration might bring extra sources for innovation into these lagging areas, and can help them catch up and consequently decrease the regional inequalities in patenting [19, 20]. However, the spatial effect of international collaborations does not seem to last long; innovation is not automatically maintained in the towns after an inventor worked for a foreign company. The patenting activity is only periodic in isolated peripheral locations and only those big towns innovate permanently that have access to both international and domestic sources.

The innovation capacity of regions highly depends on the policy mix [57] and thus our findings have important policy implications. The collaboration with international partners has been in the focus of national and regional CEE innovation policy since 2004 when these countries have joined the EU [28, 58]. However, the efficiency of CEE innovation policies is questionable at best [36, 37] and should be improved according to the recent EU Cohesion Policy that aims for sustainable and inclusive local economies by strengthening innovation [38]. Taken our findings together, policies should focus more on the synergy between international and domestic collaboration. For example, special attention should be paid to the strengthening of domestic CEE innovation in those technological sectors that internationally active inventors are working in, so that learning from foreign colleagues can create higher potential for local spillovers. Furthermore, inventors with international experience and located in peripheral locations should be helped in building connections with other CEE inventors and especially with inventors in CEE cities. A tighter network of inventors might enable a better use of innovation sources, in which central locations can be of high importance. Certainly, further research is needed to identify the specific tools for improving knowledge spillovers and for decreasing the volatility of the spatial effect of international collaborations. In doing this, one might take advantage of the dataset that we used in this paper and made available on the following link: http://datadryad.org/review?doi=doi:10.5061/dryad.5c820.

## Supporting Information

S1 Appendix Description of data cleaning. (PDF)

S2 Appendix Technological change. (PDF)

S1 Fig The global map of USPTO patenting collaboration of CEE countries 1981–1996. (TIF)

S2 Fig The global map of USPTO patenting collaboration of CEE countries 1996–2010. (TIF)
