# Supplementary material for: International Collaboration and Spatial Dynamics of US Patenting in Central and Eastern Europe 1981-2010
Source: PLoS One. 2016 Nov 15;11(11):e0166034. doi: 10.1371/journal.pone.0166034 (PMC5112948; doi:10.1371/journal.pone.0166034)
Supplement: S1 Appendix — (PDF) [file pone.0166034.s001.pdf]

Supporting Information 1. Data collection and cleaning

The database of the USPTO contains all patent data since 1790 and patents are retrievable as image files since then and after 1976 also as full text. The HyperText Markup Language (HTML) format allows us to study patents in considerable detail. One can, for example, search with names of countries, states, or city addresses in addition to the issue and/or application dates of the patents under study or classifications at the ‘Advanced Search’ engine of the USPTO database of granted patents at http://patft.uspto.gov/netahtml/PTO/search-adv.htm or patent applications at http://appft.uspto.gov/netahtml/PTO/search-adv.html. A set of dedicated routines download and organize the data into a relational database that contains patent characteristics (e. g. technological field, total number of citations), and inventor and assignee data (e.g. name and settlement level location of inventors and firms). These routines are open source, thus can be downloaded and further instructions can be found at http://www.leydesdorff.net/software/patentmaps/index.htm.

For the sake of the recent paper, we collected USPTO patents with at least one inventor in Poland, Hungary, Czechoslovakia and the successors of the latter country the Czech Republic and Slovakia, for the 1981-2010 period using the search string ‘icn/(cs OR cz OR pl OR sk OR hu) and isd/1981$$->2010$$’ on August 5, 2013. The download recalled 5,777 patents.

The publicly available data contains errors that have to be cleaned carefully. Our data cleaning focused on identifying the main technological field of patents, the names of the assignees, and the addresses of both the CEE and non-CEE assignees and CEE inventors. The location data of non-CEE inventors was not cleaned, because we do not used it in the paper.

The dataset contains the full codes for technological fields according to the Cooperative Patent Classification (CPC) that is the harmonized classification system based on the existing former classifications of ECLA (European Classification) and USPS (United States Patent Classification). One can find detailed description of the classification system at http://www.cooperativepatentclassification.org. CPC contains nine main classes identified by the first digit of the CPC code ranging from A to H, and an additional Y class; the latter was not present in our dataset. A patent can have more CPC codes and these can refer to more than one main class. We identified the main technological field of patents by taking the most frequent main class appearing in its technological field description.

Identical assignees were often recorded under multiple names, which stemmed from (1) unusual letters or typographical errors due to various language usage, and (2) divergent notation of company forms (e.g. ltd and l.t.d. cannot be considered identical). Therefore, assignee names were unified by changing all the characters into capitals and removing full stops, commas, semicolons, and further typo errors like double spaces. Subsequently, divergent formats due to different language use were unified (for example when the same university was recorded in English and Polish as well in distinct patents). Finally, the data contains institutes and their sub-institutes as different assignees; these are sometimes located in a different city (e.g. the Hungarian Academy of Sciences in Budapest has its sub-institute Biological Research Centre of the Hungarian Academy of Sciences in Szeged, 170 km from

Budapest). The remaining errors were incorrect fillings of the patents such as country or street names instead of the names of the cities, which could not be corrected and therefore were deleted from the data.

The typographical errors in the addresses were corrected by putting each assignee and inventor locations on GoogleMaps and the different formats were unified. For example, ‘Praha’, ‘Praza’ and ‘Raha’ in the Czech Republic were changed into ‘Prague’. Some of the country codes were changed during the period 1980-2010 for reasons like the dissolution of Czechoslovakia (CS) into Czech Republic (CZ) and Slovakia (SK) in 1993. In these cases country codes are only indicated as they exist currently based on the ISO 3166 standard two-digit codes at https://www.iso.org/obp/ui/#search. There were several addresses where only the country or the street name was given instead of the city names, so they were not identifiable for the map application. In these cases the headquarters of the assignees were searched manually on the internet by their names and countries. Inventors’ addresses were searched by their names, countries and the assignees of the patent on which they worked assuming if these parameters match, they are the same person. In many cases other patents were found on different sites where the address was correctly given in a more detailed format. The thorough cleaning enabled us to identify the location of most assignees and inventors.

Our remaining concern regarded the fact that settlements around large cities are recorded as separate towns in the data; however, inventors are likely to commute to the cities from the agglomeration. Therefore, we recoded those settlements that belonged to large agglomeration areas according to the following criteria. (1) Capitals, industrial and county centers have been re-coded to agglomerations. (2) If a bypass route surrounds a large city, those settlements (sometimes district names, small villages or towns) that are within that route were re-coded to the agglomeration. (3) In the case of European locations, CEE and non-CEE locations likewise, we used a 10 km radius from the city centre for supplementing the bypass ring if there was no such route found. (4) In the case of US locations, we used a 15-20 km radius, because people travel bigger distances by car and also because the usual radius of ring roads is broader in the USA than in Europe (see for example the approximately 15-20 km circle for Richmond, VA). Additionally, cities in colossal agglomerations such as New York were re-coded to the superior city even if they were remarkably further than that 10 km ring. The geo-coordinates of relevant cities have been collected by the GSP Visualizer (http://www.gpsvisualizer.com//geocoder/) and later corrected manually using GoogleMaps.

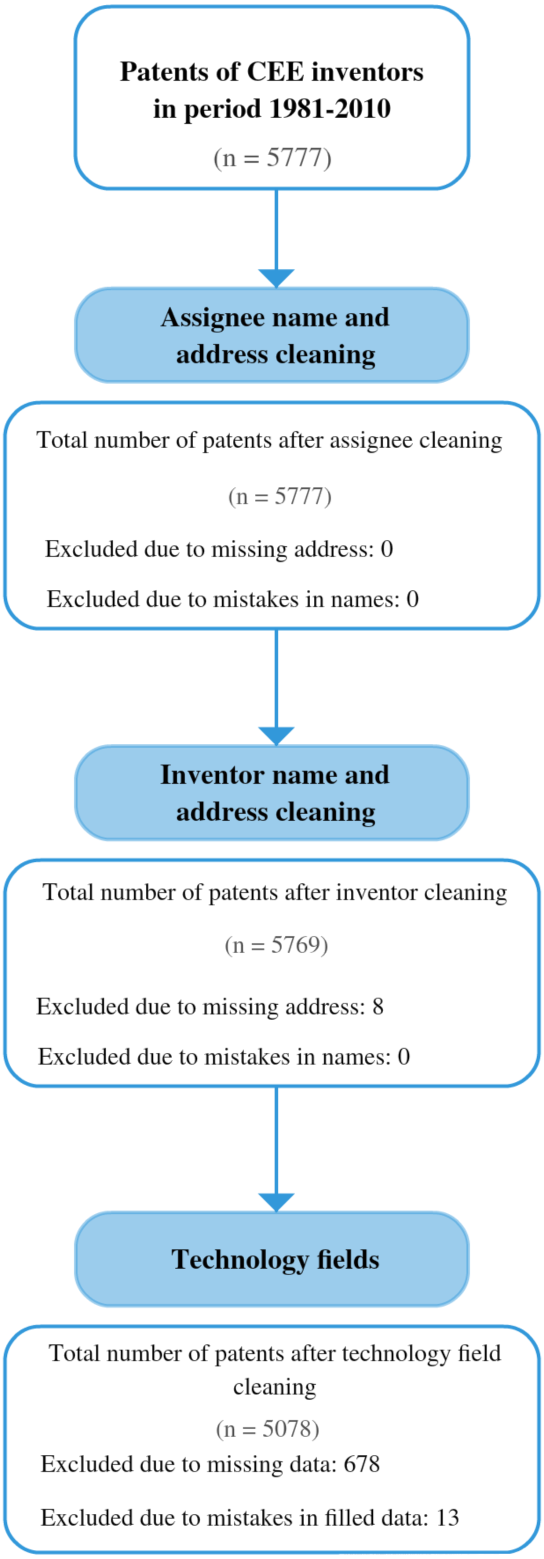

Not all the data could be cleaned and therefore we had to exclude the patents with uncertain information. The exclusion criteria and process is illustrated in Figure I. The addresses of assignees were all recognizable; thus, no patent had to be removed from the database due to incorrect filling. However, addresses of inventors were of worse quality. Focusing only on determining CEE inventors' cities of residence, 8 patents had to be deleted from the database

resulting from errors in addresses. The technological fields caused the biggest cut to the database: data on technological classification was missing in the case of 678 patents and contained error in the case of 13 patents. These patents were excluded from the database. As a result, the data contains 5,078 patents from 1,570 assignees located in 47 countries and 11,405 inventors located in 57 countries.

In the final step, we identified the geo-coordinates of assignees and CEE inventors based on the cleaned name of towns. In the last step, we matched NUTS3 region code and population size to every CEE town in our data from a publicly available EUROSTAT database that one can assess at

http://ec.europa.eu/eurostat/web/nuts/correspondence-tables/postcodes-and-nuts.
