# Supplementary material for: International Collaboration and Spatial Dynamics of US Patenting in Central and Eastern Europe 1981-2010
Source: PLoS One. 2016 Nov 15;11(11):e0166034. doi: 10.1371/journal.pone.0166034 (PMC5112948; doi:10.1371/journal.pone.0166034)
Supplement: S2 Appendix — (PDF) [file pone.0166034.s002.pdf]

Supporting Information 2. Technological change

In order to provide detailed information regarding the nature of technological change in CEE patenting and the role of foreign-controlled innovation, we break the data into 5-year periods and count the patents by technological classes and types of assignees (Table I). The P values of the chi-squared test are reported in Figure 3A of the main text.

Table I. Number of patents by technological classes, 5-year periods, and types of assignees

	1981-1985		1986-1990		1991-1995		1996-2000		2001-2005		2006-2010	
	Non-CEE	CEE	Non-CEE	CEE	Non-CEE	CEE	Non-CEE	CEE	Non-CEE	CEE	Non-CEE	CEE
A	10	159	15	155	45	65	90	41	130	66	95	64
	(9,4)	(159,6)	(20,2)	(149,8)	(48,4)	(61,6)	(93,1)	(37,9)	(144,3)	(51,7)	(123,9)	(35,1)
B	2	102	9	87	16	30	41	21	81	28	64	24
	(5,8)	(98,2)	(11,4)	(84,6)	(20,2)	(25,8)	(44,1)	(17,9)	(80,2)	(28,8)	(68,5)	(19,5)
C	20	285	29	206	100	129	146	73	164	147	88	46
	(17,0)	(288,0)	(27,9)	(207,1)	(100,7)	(128,3)	(155,7)	(63,3)	(228,9)	(82,1)	(104,4)	(29,6)
D	0	40	1	19	8	15	10	35	32	12	79	27
	(2,2)	(37,8)	(2,4)	(17,6)	(10,1)	(12,9)	(32,0)	(13,0)	(32,4)	(11,6)	(82,6)	(23,4)
E	1	17	2	11	0	1	3	7	1	5	3	1
	(1,0)	(17,0)	(1,5)	(11,5)	(0,4)	(0,6)	(7,1)	(2,9)	(4,4)	(1,6)	(3,1)	(0,9)
F	1	62	1	43	6	17	20	16	71	21	51	12
	(3,5)	(59,5)	(5,2)	(38,8)	(10,1)	(12,9)	(25,6)	(10,4)	(67,7)	(24,3)	(49,1)	(13,9)
G	10	68	18	56	22	9	90	15	200	32	263	37
	(4,3)	(73,7)	(8,8)	(65,2)	(13,6)	(17,4)	(74,6)	(30,4)	(170,7)	(61,3)	(233,7)	(66,3)
H	1	29	6	25	19	9	116	2	227	14	245	41
	(1,7)	(28,3)	(3,7)	(27,3)	(12,3)	(15,7)	(83,9)	(34,1)	(177,4)	(63,6)	(222,8)	(63,2)
Chi2	15,56		19,71		22,22		121,33		157,25		71,12	
P	0,03		0,01		0,00		0,00		0,00		0,00	

Note: Expected values under the validity of the null hypothesis in parantheses.

To test whether technological change of CEE patenting was significant over the full 1981-2010 period, we apply the repeated ANOVA method. We chose a model in which the number of patents by technology classes is described by a between-subject effect that is the type of assignee (CEE equals 1 in the case of CEE assignees and 0 in the case of non-CEE asignees) and a within-subject factor that is constituted by the 5-year periods. The error term of the between-subject effect the technological class nested in CEE; while the error term of the within-subject factor is the residual of the model.

The model in Table II. suggest a significant effect of the within-factor and the interaction of within-factor and the between-subject effect because the p-values of the period variable and the CEE#period interaction is lower than 0.01.

Table II. The significance of technological change

Source	Partial SS	df	MS	F	Prob > F
Model	280552.571	25	11222.103	7.66	0.000
CEE	238.115	1	238.116	0.02	0.887
tech | CEE	158655.810	14	11332.558		
period	26329.824	5	5265.965	3.59	0.006
CEE#period	101970.460	5	20394.092	13.91	0.000
Residual	99671.386	68	1465.756		
Total	380223.957	93	4088.42965		

Note: Number of obs = 94; Root MSE = 38.285; R-squared=0.738; Adj R-squared = 0.642

However, repeated ANOVA assumes that the within-subject covariance structure is compound symmetric and the violation of the assumption the p-values may be biased. Therefore, we computed p-values for conservative F-tests that report correct p-values even if the data do not meet the compound symmetry assumption. Results in Table III. illustrate that the CEE#period interaction is still significant but the period effect is only significant at the 5% level in case of the Huynh-Feldt and Greenhouse-Geisser tests but looses significance in case of Box’s conservative F-test.

Table III. The significance of technological change under conservative F -tests

			Prob > F			
Source	df	F	Regular	H-F	G-G	Box
period	5	3.59	0.0061	0.0383	0.0488	0.0795
CEE#period	5	13.91	0.0000	0.0000	0.0002	0.0023
Residual	68					

Huynh-Feldt epsilon= 0.4227; Greenhouse-Geisser epsilon = 0.3488; Box's conservative epsilon = 0.2000

The strongly significant effect of the CEE#period interaction and the loosely significant effect of period main effect suggests a significant technological change over 1981-2010 in CEE patenting, in which the foreign-controlled innovation played a major role.
