# Supplementary figures and images for: International Collaboration and Spatial Dynamics of US Patenting in Central and Eastern Europe 1981-2010
Source: PLoS One. 2016 Nov 15;11(11):e0166034. doi: 10.1371/journal.pone.0166034 (PMC5112948; doi:10.1371/journal.pone.0166034)

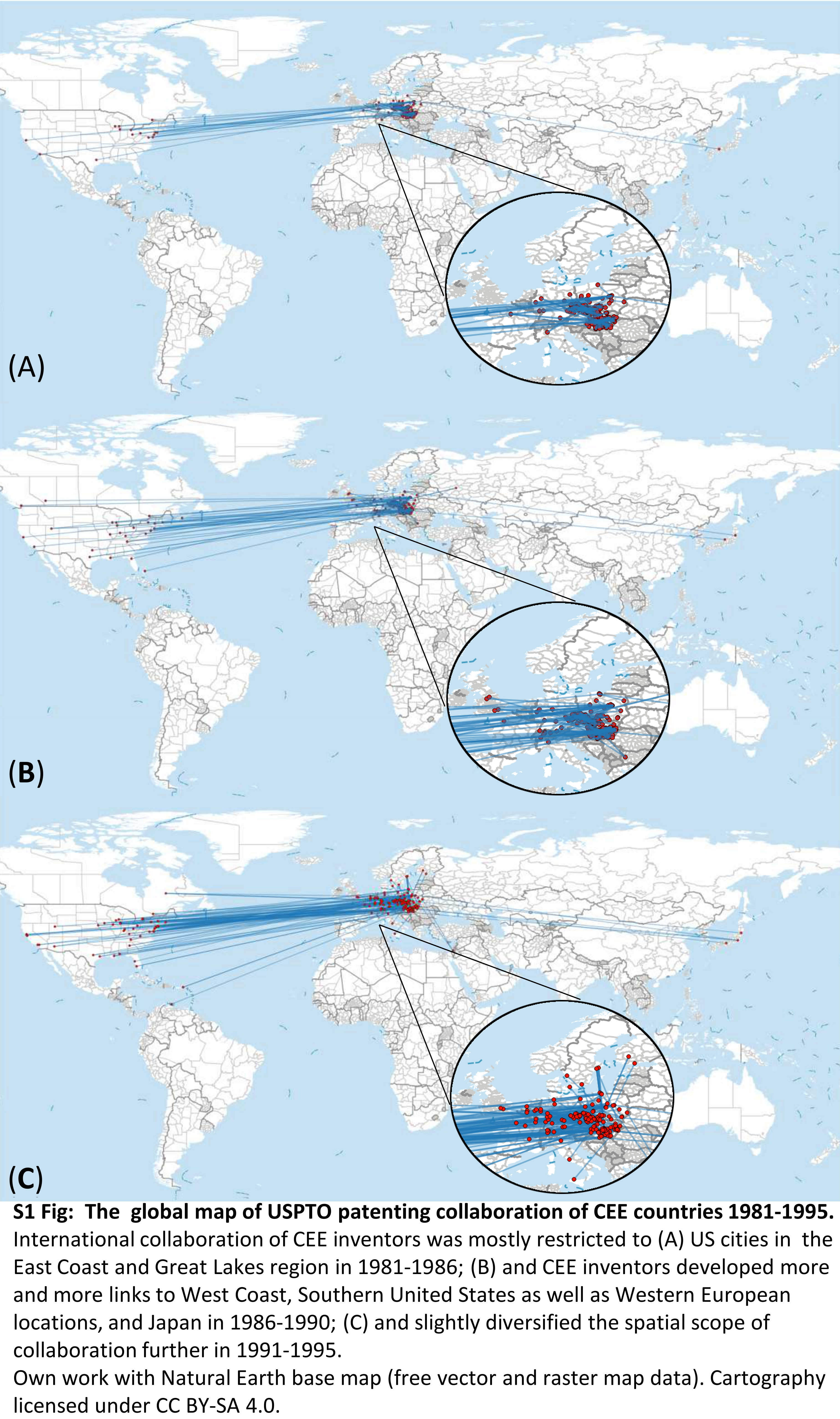

Supplement: S1 Fig — (TIF) [file pone.0166034.s003.tif]

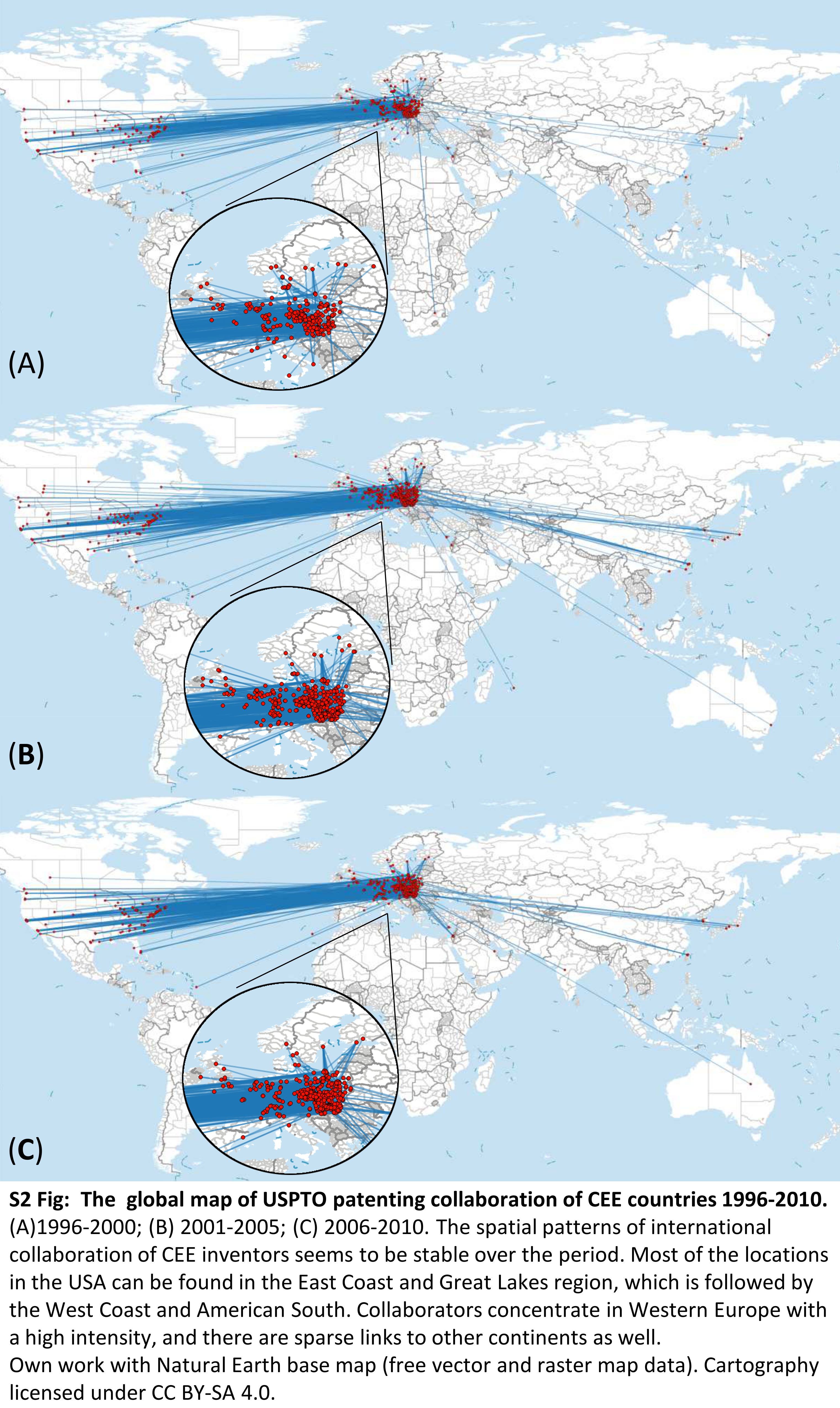

Supplement: S2 Fig — (TIF) [file pone.0166034.s004.tif]
